# A Two-Photon Fluorescent Probe for Lysosomal Thiols in Live Cells and Tissues

**DOI:** 10.1038/srep19562

**Published:** 2016-01-22

**Authors:** Jiangli Fan, Zhichao Han, Yao Kang, Xiaojun Peng

**Affiliations:** 1 State Key Laboratory of Fine Chemicals, Dalian University of Technology, Dalian, 116024, China

## Abstract

Lysosome-specific fluorescent probes are exclusive to elucidate the functions of lysosomal thiols. Moreover, two-photon microscopy offers advantages of less phototoxicity, better three dimensional spatial localization, deeper penetration depth and lower self-absorption. However, such fluorescent probes for thiols are still rare. In this work, an efficient two-photon fluorophore 1,8-naphthalimide-based probe conjugating a 2,4-dinitrobenzenesulfonyl chloride and morpholine was designed and synthesized, which exhibited high selectivity and sensitivity towards lysosomal thiols by turn-on fluorescence method quantitatively and was successfully applied to the imaging of thiols in live cells and tissues by two-photon microscopy.

Low molecular weight thiols, such as cysteine (Cys), homocysteine (Hcy) and glutathione (GSH), are components of many peptides that play key roles in the pathogenesis of serious diseases and disorders^1^. For example, abnormal levels of Cys result in hair depigmentation, edema, liver damage, slow growth in children, and so on^2^. High concentrations of homocysteine (Hcy) are linked with cardiovascular and Alzheimer’s disease^3^. GSH, a tripeptide, is the most abundant intracellular non-protein thiol and plays a critical role towards controlling oxidative stress therefore maintaining redox homeostasis, which is crucial for cell growth and normal cell function^4^. In light of the aforementioned examples, efficient monitoring of the level of thiols in live cells and tissues is crucial for identifying abnormal biological processes and risk towards diseases.

Modern analytical techniques for the selective detection of thiols in a chemical system includes potentiometry^5^, high-performance liquid chromatography^6^,^7^, and UV/Vis absorption spectrophotometry^8^. Among these, fluorescent detection has attracted much deserved attention due to the high sensitivity, operational simplicity and potential use towards intracellular bioimaging^9^,^10^,^11^,^12^,^13^. A variety of fluorescent probes utilize the high nucleophilic reactivity or transition metal-affinity of thiol groups, which involves a specific reaction between the probe and thiols (e.g., Michael addition^14^,^15^, cyclization with aldehyde^16^,^17^, cleavage reaction^4^,^18^,^19^, nucleophilic substitution^20^,^21^,^22^,^23^,^24^, disulfide exchange reaction^25^,^26^,^27^, deprotection of 2,4-dinitrobenzene-sulfonyl (DNBS) and others^28^,^29^,^30^,^31^,^32^). The DNBS thiol detection group is of particular interest due to its unique sensitivity and high reactivity toward thiolate anions affording an OFF–ON signal response.

As an essential organelle, the lysosome, within approximately 50 hydrolases, is responsible for the degradation and recycling of macromolecules^33^,^34^. Thiols are closely associated with proteolysis in the lysosome which reduces disulphide bonds^35^. For example, stabilization of lysosome membranes may involve GSH, whereas Cys is an effective stimulator of albumin degradation in liver lysosomes^36^. Therefore, fluorescent probes that respond to thiols specifically within the lysosome are of particular interest to elucidate organelle functionality. Despite modern achievements with lysosome fluorescent probes, detecting thiols in some special organelles is still very challenging^14^,^37^,^38^,^39^,^40^,^41^,^42^. The traditional one-photon microscopy imaging technique (OPM), that observes the response of the fluorescent probe with thiols, has been favorably substituted with a two-photon microscopy (TPM) due to lower photo-toxicity, improved three dimensional spatial localization, deeper penetration and decreased self-absorption events^43^,^44^. Thus an attractive approach for the detection of thiols in organelles, deep inside the tissues, is through the use of two-photon microscopy (TPM).

Herein, we present a lysosome fluorescent probe based on an efficient two-photon fluorophore, 1,8-naphthalimide^45^ coupled with a popular DNBS as the transponder. We investigated its optical properties and response towards various amino acids. The results suggested that it possesses impressively high selectivity towards thiols and it can be used effectively as an indicator to monitor the level of thiols in lysosomes.

## Results and Discussion

### Design and synthesis

**Figure 1:**
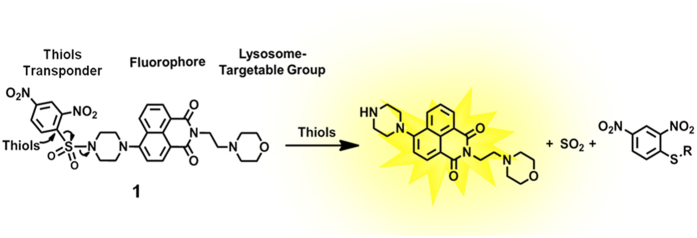
Chemical structure of 1 and reaction of 1 with thiols.

The lysosome-specific fluorescent probe was designed with three main components: 1) a lysosome targeting head-group; 2) a central fluorescent molecule; and 3) a thiol responsive tail-group. The head-group consisting of morpholine is a widely accepted lysosome directing group^46^,^47^,^48^,^49^, whereas 4-amino-1,8-naphthalimide, the central component, has fluorophore properties due to its ICT(intramolecular charge transfer) states and large two-photon absorption cross section^50^. We chose 2,4-dinitrobenzenesulfonyl (DNBS) for its known reactivity with thiols and utilization as a detector in other systems^4^. As there is a photo-induced electron transfer (PET) between DNBS and naphthalimide, the fluorescence is essentially quenched. Once the DNBS is hydrolyzed by thiols, inhibition caused by the PET process is terminated thereby restoring the observed fluorescence of naphthalimide (Fig. 1).

**Figure 2:**
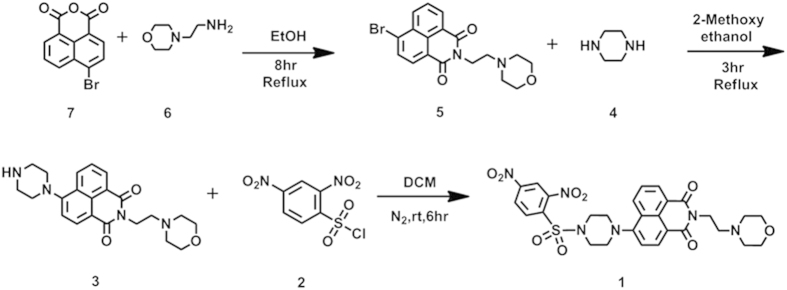
Synthesis of lysosome-targetable thiols 1.

The synthetic route utilized to prepare **1** is illustrated in Fig. 2. Briefly, 4-bromo-1,8-naphthalic anhydride (7)was treated with 2-morpholinoethanamine (**6**) in ethanol to afford **5** 51. Subsequent ammonization with piperazine (**4**) generated the intermediate **3** 52, which then was reacted with 2,4-dinitrobenzenesulfonyl chloride (DNBS-Cl, **2**) in the presence of Et_3_N to give **1** with a yield of 75.0%^4^. The structures were well characterized by proton and carbon nuclear magnetic spectroscopy (^1^H-, ^13^C-NMR) and high-resolution mass spectra (HRMS). Detailed synthetic procedures and structure characterizations are provided in the ESI.

### Optical properties

**Figure 3:**
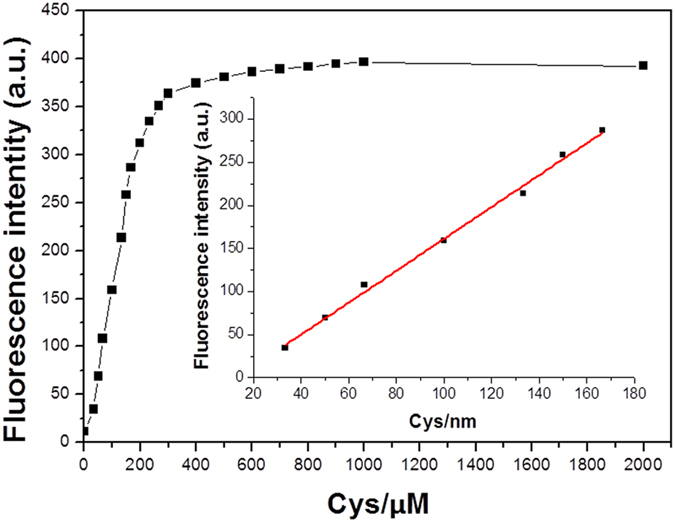
Fluorescence spectra changes of 1 (10 μM) upon addition of different concentrations of Cys. Each spectrum was obtained 10 min after Cys addition. λ_ex_ = 400 nm, slit width (5, 5). Insert: Linear relationship of fluorescence intensity at 540 nm as a function of Cys concentration, y = −23.61 + 1.8488 [Cys], with R^2^ = 0.9956.

The fluorescent titration of **1** (10 μM) upon addition of Cys (0–200 equiv), prepared in DMSO-HEPES (DMSO: dimethyl sulfoxide, HEPES: 4-(2-hydroxyethyl)-1-piperazineethanesulfonic acid) (10 mM, pH 7.4, 3:7, v/v) was investigated (Fig. 3). At first, the probe **1** exhibits extremely weak fluorescence (*Φ* = 0.015). As would be expected, the addition of Cys resulted in an elevated fluorescence emission signal which became stronger with the increasing concentration of Cys. A strong linear relationship between the fluorescence intensity and concentration of Cys (ranging from 30 to 160 μM), was observed. The standard deviation (**σ**), according to the fluorescence response, was determined to be 0.1591. Therefore, the detection limit for Cys reaction with **1** was calculated, from the signal/noise ratio (S/N = 3), to be 2.6 × 10^−7^ M. Under these same conditions, **1** exhibited different fluorescence responses to GSH and Hcy with detection limits of 2.41 × 10^−6^ M and 4.87 × 10^−6^ M, respectively (Figs S2 and S3).

**Figure 4:**
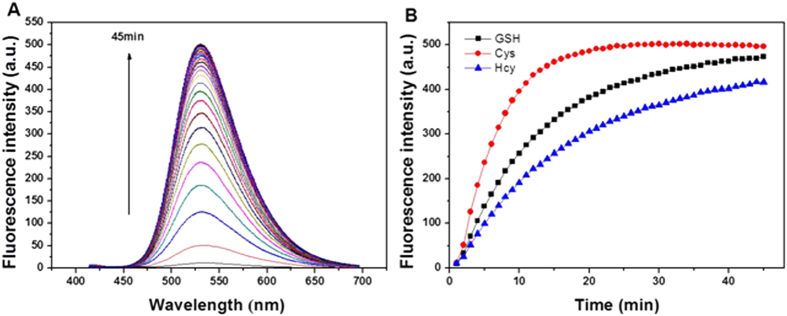
(**A**) Time-dependent emission spectra (λ_ex_ = 400 nm) of **1** (10 μM) upon treatment with Cys (100 equiv) in DMSO-HEPES (10 mM, pH 7.4, 3:7, v/v) at 37 °C. Spectra were recorded every min (0–45 min). (**B**) Changes in fluorescence intensity at 540 nm in the presence of Cys, Hcy and GSH (100 equiv) as a function of incubation time, respectively.

The fluorescence response of **1** by thiols was tested and illustrated in Fig. 4 and supplementary information (S4 and S5). The thiols, Cys in particular, were prepared in DMSO-HEPES (10 mM, pH 7.4, 3:7, v/v) and added to the **1** solution at a temperature of 37 °C. The spectra indicates that a time-dependent change occurred upon the addition of Cys (100 equiv.) to the solution of **1** (10 μM). Within 10 minutes, an obvious shift in the fluorescence intensity was observed (Fig. 4A) in which it took about 20 minutes to attain the equilibrium state (Fig. 4B). And it took about 45 minutes to attain an equilibrium state for both Hcy and GSH at lower fluorescence intensities (Fig. 4B). Overall, the emission intensity of **1** was elevated by a 74-fold, 63-fold and 60-fold over its initial state and the fluorescence quantum yields increased to 0.52, 0.45 and 0.43 for Cys, GSH and Hcy, respectively, from the initial quantum yield of 0.015.

**Figure 5:**
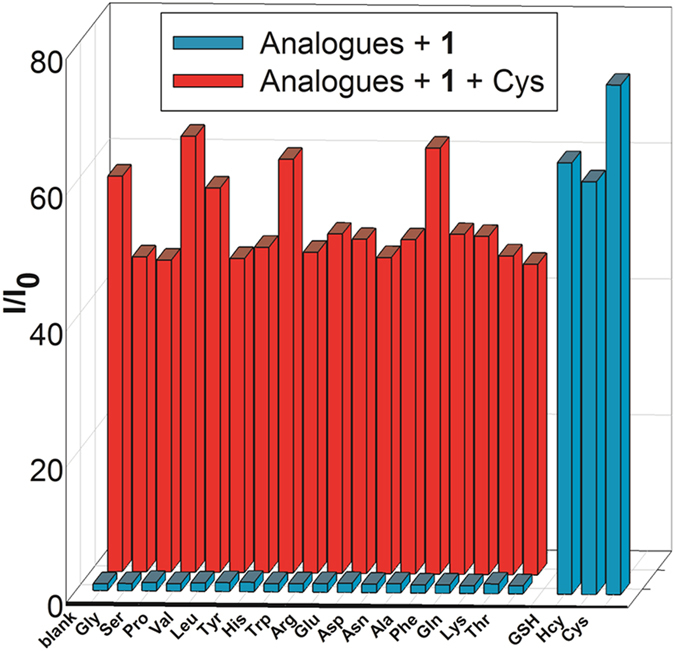
Fluorescence responses of 1 (10 μM) to various analogues (Cys, Hcy, GSH and 17 amino acids at 100 μM). Blue bars represent **1**+ analogues; red bars represent **1**+ Cys in the presence of other analogues (100 μM). Data was collected at 45 min after addition of each analogue in DMSO-HEPES (10 mM, pH 7.4, 3:7, v/v) at 37 °C.

The chemical selectivity of **1** (10 μM) toward thiols versus amino acids was tested by means of measuring the time-dependent fluorescent changes. Solutions of 17 amino acids (Fig. 5; three letter codes utilized in x-axis) and the 3 thiols (i.e., Cys, Hcy, and GSH) were screened against aliquots of the **1** solution individually. The figure illustrates that no significant changes were detectable, beyond an incubation period of 45 minutes, with various amino acids. However, an obvious increase in the fluorescence intensity was observed only when thiols were added, which indicated that **1** could be used to selectively detect these three thiols. In addition, competition experiments were conducted in which each thiol analogue was combined with an amino acid in the presence of probe **1**. Significantly, the results showed that fluorescent detection of thiols (exemplified by Cys in Fig. 5) by **1** in the presence of various amino acids was still effective. Therefore, **1** has a high selectivity for thiols.

The effect of pH on the photophysical properties of **1** was also explored (S7). The probe was quite stable and unresponsive to a pH range of 3–13. However, the addition of GSH, Cys, or Hcy (100 μM) afforded a solutions pH from 6.0 to 7.5 and increased the overall fluorescence, which clearly demonstrated that the fluorescence response of **1** in the physiological pH range was due to the presence of thiols.

### Bioimaging in live cells

**Figure 6:**
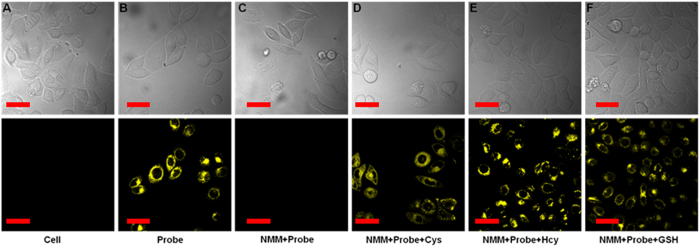
Top Row: Optical microscopy images of HeLa cells (**A**) untreated; (**B**) incubated with 1 (5 μM) for 20 min; (**C**) pretreated with NMM (1 mM) followed by incubation with 1; and (**D–F**) pretreated with NMM, then treated with Cys, Hcy, or GSH (100 μM), respectively, and finally incubated with 1. Bottom Row: Fluorescent emission images of the above cell lines. Two-photon excitation was provided at 805 nm with fs pulses, and the TPEF were collected at 520–560 nm. Scale bar: 30 μm. Cells are shown representative images from replicate experiments (n = 5).

To validate the practical utility of **1** in biological samples, the imaging of intracellular thiols was tested in live cells. First, we tested the cell permeability and intracellular thiols sensing ability of **1**. As shown in Fig. S9A, the HeLa cells showed no fluorescence activity. However, when HeLa cells were incubated with **1** (5 μM) at 37 °C for 20 min, the cells started to show a strong yellow fluorescence. This verified that **1** could permeate into cells and react with resident thiols to generate distinguishable fluorescence images. For a control experiment, HeLa cells were pretreated with an excess (1 mM) of thiol-reactive N-phenylmaleimide (NMM, a known thiol-blocking agent)^53^ and then incubated with **1** for 20 min, no fluorescence was observed (Fig. S9C). With the addition of GSH, Cys or Hcy (100 μM) to these NMM–pretreated HeLa cells, and subsequent incubation with **1** (5 μM) for another 20 min, a remarkable increase in yellow emission (Fig. S9D–F) was observed. Bright field image measurements confirmed that the cells were viable throughout the imaging studies (Fig. S9). To gain further insight into **1**’s efficacy in live cells, the probe was subjected to live MCF-7 cells and found to be fluorescent (Fig. S10). The aforementioned experiments confirmed the specificity of **1** towards thiols over other biomolecules in live cells. We then tested the two-photon imaging ability of **1** to detect thiols in HeLa and MCF-7 cells by using a higher excitation wavelength, 805 nm (scanning lambda mode), and collected the subsequent emission signals at 520 and 560 nm, respectively. The results were similar to the lower excitation and may be viewed in Fig. 6 as well as in ESI (S8).

**Figure 7:**
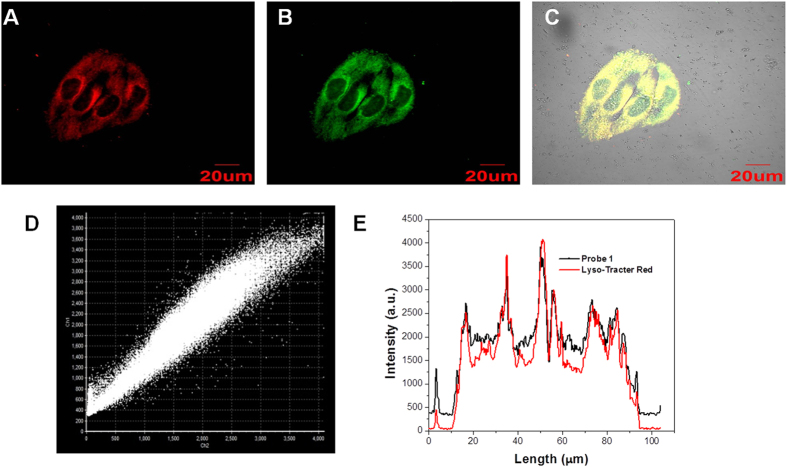
Co-localization experiments using 1 to lysosomes in HeLa cells. Cells were stained with (**A**) **1** (5 μM) for 20 min at 37 °C and (**B**) Lyso-Tracker Red (1.0 μM). (**C**) overlay of (**A**,**B**). (D) Intensity correlation plot of stain **1** and Lyso-Tracker Red. (**E**) Intensity profile of regions of interest (ROI) across HeLa cells. The excitation wavelengths were 405 nm (**1**) and 559 nm (Lyso-Tracker Red). The fluorescence was collected at 510–540 nm (**1**) and 580–600 nm (Lyso-Tracker Red).

To explore the subcellular localization of **1**, a commercially available lysosome-localizing dye Lyso-Tracker Red was used for a co-localization study (Fig. 7 and Figs S11 and S12). Co-localization was quantified using Pearson’s sample correlation factors (Rr). The intensity of correlation plots (Fig. 7D) revealed a high Pearson’s coefficient (0.97), confirming that **1** was a true lysosome-targeted probe. The cytotoxicity of **1** was determined by the MTT assay. Various concentration of **1** (1, 5, 10, 20, 30 and 50 μM) were used to determine the toxicity level of the **1** towards MCF-7. The result indicated cells were not affected by incubation with **1** for 24 h (Fig. S13).

**Figure 8:**
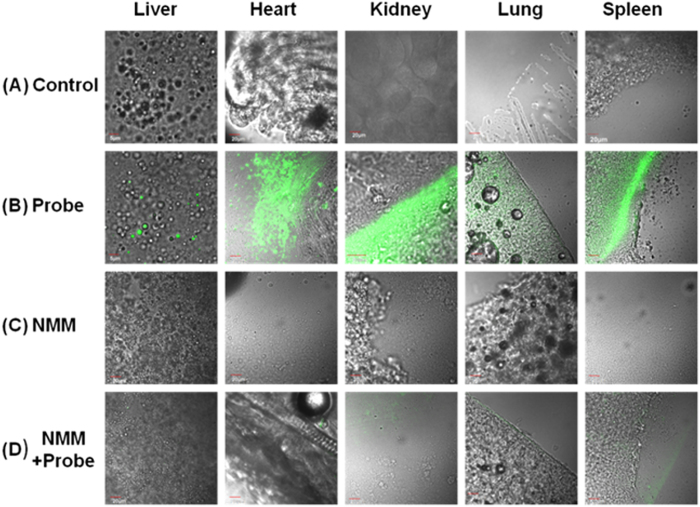
TPM images at a depth of ~120 μm of tissues from mouse intravenously injected with 1 (10 μM) or NMM (20 mM). Fluorescence images of liver, heart, kidney, lung and spleen. (**A**) Dissected from mouse no injected with **1**, (**B**) mouse injected with **1**, (**C**) mouse injected with NMM, and (**D**) mouse injected with **1** after pre-injection with NMM. The TPM images were collected at 520–560 nm upon excitation at 805 nm with fs pulse.

The achievement in the cell experiments encouraged us to investigate the utility of **1** in tissue imaging. ICR mice were selected as our model and were given intravenous injections of buffer solution containing **1** (10 μM). After 20 min, they were observed by two-photon excitation. The mouse was dissected to isolate various tissues, remarkable fluorescence images were acquired from liver, heart, kidney, lung, and spleen (Fig. 8B). Another mouse was preinjected with NMM (20 mM, 200 μL in HEPES buffer solution). After 30 min, the mouse was injected with **1** (10 μM). The mouse was dissected to isolate various tissues too (Fig. 8D), low fluorescence intensity was observed when the mice were pretreated with NMM. Significantly, the tissue slices could be clearly visualized by yellow fluorescence at 20–140 μm depth (Fig. S14).

### Reaction Mechanism

Elucidating the mechanism of the fluorescence “off-on” process is vital, for clarifying the optical properties. We supposed that **1** was possibly involved in the reaction shown in Fig. 1. To further demonstrate the recognition mechanism of **1** for thiols, we check the mass spectrum. **1** showed a peak at 625.1712 (m/z) before adding Cys (Fig. S25). A new peak at 395.2028 (m/z) (Fig. S22) was assigned to the cleavable product **3** (Fig. 1). Moreover, the UV/Vis absorption spectroscopic of **1** changes showed that addition of thiols (100 equiv) to a solution of **1** (10 μM) in DMSO-HEPES (10 mM, pH 7.4, 3:7, v/v) led to an increase in absorbance at 375 nm (Fig. S15a). Similarly, mixing **1** with a thiol led to production of a fluorescence emission band with a maximum at 525 nm (Fig. S15b). Consequently, more detailed analysis of the structure and electron density was performed in an attempt to gain insight into its recognition mechanism. The calculated structures of **1** and **3** were optimized and their frontier molecular orbital energies were calculated by using Gaussian 09 [DFT at the B3LYP/6–311G(d,p)]^54^. As shown in Figure S16, the demonstration of HOMO and LOMO levels support a possible photoinduced electron transfer (PET) process in **1**. The piperazine ring in **1** and compound **3** showed a table chair conformation. For **1**, the electron density mainly focused on 2,4-dinitrobenzenesulfonyl chloride unit in its HOMO orbital, the electron transfer occurs to the naphthalimide unit, this diminished the fluorescence of the original fluorophore, resulting in fluorescence being “switched off”. While the cleavable product **3** was only involved in the part of electron transfer from piperazine ring to naphthalimide, it resulted in elimination of the PET fluorescence quenching process. Therefore, the cleavable product **3** was fluorescent. Moreover, the calculated HOMO-LUMO energy gaps of **1** (1.76 eV), which was lower in comparison to **3**.

## Conclusion

In summary, we developed a two-photon fluorescent probe **1** based on 1,8-naphthalimide by introducing DNBS into 4-position of naphthalimide with piperazine as a linker and morpholine as lysosome directing group. Due to the reaction of thiols with DNBS, a large fluorescence increase was obtained with emission centered at 540 nm in aqueous solution. Cell-imaging and tissue experiments reveal that **1** is a new lysosome-specific and two-photon probe for thiols. The results of this effort strongly suggest that **1** can serve as an effective tool for studies probing cellular lysosome functions that are related to thiols.

## Additional Information

**How to cite this article**: Fan, J. *et al*. A Two-Photon Fluorescent Probe for Lysosomal Thiols in Live Cells and Tissues. *Sci. Rep*. **6**, 19562; doi: 10.1038/srep19562 (2016).

## Supplementary Material

Supplementary Information
