# Supplementary material for: A Two-Photon Fluorescent Probe for Lysosomal Thiols in Live Cells and Tissues
Source: Sci Rep. 2016 Jan 22;6:19562. doi: 10.1038/srep19562 (PMC4726187; doi:10.1038/srep19562)
Supplement: Supplementary Information [file srep19562-s1.pdf]

Supplementary Information

A Two-Photon Fluorescent Probe for Lysosomal Thiols in Live Cells and Tissues

Jiangli Fan*, Zhichao Han, Yao Kang, Xiaojun Peng

State Key Laboratory of Fine Chemicals, Dalian University of Technology, Dalian, 116024, China

* Corresponding Author: Jiangli Fan Email: fanjl@dlut.edu.cn Tel/Fax: +86-411-84986327

Contents

1. Materials and general methods
2. Synthesis of **1**
3. Determination of quantum yields
4. Determination of the detection limit
5. Cytotoxicity experiments
6. Live cell imaging experiments
7. Tissue imaging experiments
8. Supplementary references

Fig. S1 Absorbance and emission spectra of **1** in various solvents
Table S1 Photophysical properties of **1** in various solvents
Fig. S2 Fluorescence intensity (540 nm) of **1** upon the titration of GSH
Fig. S3 Fluorescence intensity (540 nm) of **1** upon the titration of Hcy
Fig. S4 Time-dependent fluorescence response of **1** to GSH
Fig. S5 Time-dependent fluorescence response of **1** to Hcy
Fig. S6 Selectivity experiments
Fig. S7 Effect of pH on the fluorescence intensities of **1** in the absence and presence of thiols
Fig. S8 TPM images of **1** response toward NEM, thiols and a mixture of NEM and thiols in MCF-7
Fig. S9 Fluorescence responses of **1** toward NEM, thiols and a mixture of NEM and thiols in HeLa
Fig.S10 Fluorescence responses of **1** toward NEM, thiols and a mixture of NEM and thiols in MCF-7
Fig. S11Colocalization experiment in COS-7 cells
Fig. S12 Colocalization experiment in MCF-7 cells
Fig. S13 Cell viability assay
Fig. S14 Depth imaging of **1** in tissue
Fig. S15 Absorbance and emission spectra of **1** and compound **3**
Fig. S16 Frontier molecular orbital profiles of **1** and **3**
Fig. S17-25 NMR and MS data for compounds

1. Materials and general methods

All of the solvents used were of analytic grade. **1** was dissolved in dimethyl sulphoxide (DMSO) to produce 5 mM stock solutions. Aliquots were then diluted to 10 μM with a HEPES buffer solution containing 40 mM acetic acid, phosphoric acid, and boric acid. Slight variations in the pH of the solutions were achieved by adding minimal volumes of NaOH or HCl. ^{1}H-NMR and ^{13}C-NMR spectra were recorded on a VARIAN INOVA-400 spectrometer. Chemical shifts (δ) were reported as ppm in $CDCl_3$, with TMS as the internal standard. Mass spectrometric (MS) data were obtained with HP1100LC/MSD MS and an LC/Q-TOF-MS instruments. Fluorescence measurements were performed on a VAEIAN CARY Eclipse fluorescence spectrophotometer (Serial No. FL0812-M018). Excitation and emission slit widths were modified to adjust the fluorescence intensity to a suitable range. Absorbance spectra were measured on a Perkin Elmer Lambda 35 UV/VIS spectrophotometer. All pH measurements were performed using a Model PHS-3C meter calibrated at room temperature (23 ± 2 ℃) with standard buffers of pH 7.4.

2. Synthesis of 1

Synthesis of compound 5. 4-(2-aminoethyl)-morpholine (1.30 g, 0.01 mol) was added to 4-bromo-1,8-naphthalic anhydride (2.77 g, 0.01 mol) in boiling 1,4-dioxane (50 mL) under magnetic stirring for 4h. After cooling to room temperature, the yellowish sediments were collected by filtration and then dried overnight at room temperature in a vacuum oven to give **5** as a pale yellow solid (2.72 g), yield: 70.3 %. ^{1}H-NMR [400 MHz, $CDCl_3$, δ (ppm)]: 8.65 (1H, d, *J* = 8.0 Hz), 8.57 (1H, d, *J* = 8.0 Hz), 8.40 (1H, d, *J* = 4.0 Hz), 8.04 (1H, d, *J* = 4.0 Hz), 7.86 (1H, t, *J* = 8.0 Hz), 4.35 (2H, t, *J* = 8.0 Hz), 3.68 (4H, t, *J* = 4.0 Hz), 2.72 (2H, t, *J* = 8.0 Hz), 2.59 (4H, s). ^{13}C-NMR [100 MHz, $CDCl_3$, δ (ppm)]: 163.50, 163.47, 133.16, 131.94, 131.12, 131.04, 130.52, 130.20, 128.90, 128.02, 122.99, 122.12, 67.03, 56.08, 53.81, 37.33. HR MS: m/z calcd for $C_{18}H_{17}N_2O_3Br^+$ $[M]^+$: 389.0489, found: 389.0495.

Synthesis of compound 3. Compounds **5** (194 mg, 0.5 mmol) and piperazine (500 mg, 2 mmol) were dissolved in anhydrous 2-methoxyethanol (10 mL) under nitrogen condition. After being stirred for 4 h at 120 ℃, the mixture was cooled to room temperature. Removal of solvent under reduced pressure and purification by silica gel column chromatography with dichloromethane/methanol (20:1) as the eluent generated **3** as a yellow solid (153.8 mg), yield: 78.4 %. ^{1}H-NMR [400 MHz, $CDCl_3$, δ (ppm)]: 8.60 (d, *J* = 7.9 Hz, 1H), 8.54 (d, *J* = 8.0 Hz, 1H), 8.44 (d, *J* = 9.1 Hz, 1H), 7.25 (d, *J* = 8.1 Hz, 1H), 4.35 (t, *J* = 7.0 Hz, 2H), 3.71 (t, *J* = 8.0 Hz , 4H), 3.29 (d, *J* = 9.5 Hz, 7H), 2.72 (t, *J* = 8.0 Hz, 2H), 1.27 (s, 6H). ^{13}C-NMR [100 MHz, $CDCl_3$, δ (ppm)]: 163.39, 163.94, 155.39, 132.45, 131.28, 129.97, 126.06, 123.43, 117.54, 115.44, 67.08, 56.22, 53.84, 53.23, 37.17. HR MS: m/z calcd for $C_{22}H_{26}N_4O_3^+$ $[M]^+$: 394.4721, found: 396.2080.

Synthesis of compound 1. Compound **3** (197 mg, 0.5 mmol) and Et_3N (30 mg, 0.3 mmol) were dissolved in anhydrous dichloromethane (10 mL) under an argon atmosphere. After the resulting solution was stirred for 5 min, a solution of 2,4-dinitrobenzene-1-sulfonyl chloride (133 mg, 0.5 mmol)

in dichloromethane (5 mL) was added dropwise at 0 ℃. Stirring for another 4 h at room temperature, the solvent was removed under reduced pressure and purified by silica gel column chromatography with dichloromethane/methanol (75:1) as the eluent to produce **1** as a bright yellow solid (142 mg), yield: 75.0%. ^{1}H-NMR [400 MHz, $CDCl_3$, δ (ppm)]: 8.56 (m, *J* = 8.0 Hz, 4H), 8.32 (t, *J* = 9.3 Hz, 2H), 7.71 (t, *J* = 7.4 Hz, 1H), 7.26 (d, *J* = 7.9 Hz, 1H), 4.32 (s, 2H), 3.68 (s, 8H), 3.35 (s, 4H), 2.65 (d, *J* = 50.5 Hz, 6H). ^{13}C-NMR [100 MHz, $CDCl_3$, δ (ppm)]: 164.22, 163.76, 154.46, 150.03, 148.41, 137.14, 132.78, 132.25, 131.32, 129.81, 129.38, 126.28, 123.47, 119.91, 118.17, 115.84, 66.99, 56.16 , 53.79, 52.72, 46.34, 37.12, 35.91, 31.89, 29.50, 29.35, 29.29, 27.21, 25.52, 22.67, 14.09. HR MS: m/z calcd for $C_{28}H_{28}N_6O_9S^+$ $[M]^+$: 625.6243, found: 625.1712.

3. Determination of quantum yields

The fluorescence quantum yield of **1** was determined according to the method below[1].

$$\varphi_u = \frac{(\varphi_s)(FA_u)(A_s)(\lambda_{exs})(\eta_u^2)}{(FA_s)(A_u)(\lambda_{exu})(\eta_s^2)}$$

Where φ is fluorescence quantum yield; *FA* is integrated area under the corrected emission spectra; *A* is the absorbance at the excitation wavelength; λ_{ex} is the excitation wavelength; η is the refractive index of the solution; the subscripts *u* and *s* refer to the unknown and the standard, respectively. We choose rhodamine B as standard, which has a fluorescence quantum yield of 0.49 in ethanol[2].

4. Determination of the detection limit

Calculation of detection limit was based on the fluorescence titration curve (Fig 3, S2 and 3) of **1** in the presence of thiols. The fluorescence intensity of **1** was measured by three times and the standard deviation of blank measurement was achieved. The detection limit was calculated with the following equation:

Detection limit = 3σ/k

Where σ is the standard deviation of blank measurement, k is the slop between the fluorescence intensity versus thiols concentrations.

5. Cytotoxicity experiments

Measurement of cell viability was evaluated by reducing of MTT (3-(4,5)-dimethylthiahiazo (-2-yl) -3,5-diphenytetrazoliumromide) to formazan crystals using mitochondrial dehydrogenases (Mosmann, 1983). Human breast cancer cells (MCF-7) cells were seeded in 96-well microplates (Nunc, Denmark) at a density of 1×10^5 cells / mL in 100 μL medium containing 10 % FBS. After 24 h of cell attachment, the plates were then washed with 100 μL / well PBS. The cells were then cultured in medium with 2.5 μM of **1** for 24 h. Cells in culture medium without **1** were used as the control. Six replicate wells were used for each control and test concentration. 10 μL of MTT (5 mg / mL) prepared in PBS was added to each well and the plates were incubated at 37 ℃ for another 4 h in a 5 % CO_2 humidified incubator.

The medium was then carefully removed, and the purple crystals were lysed in 200 μL DMSO. Optical density was determined on a microplate reader (Thermo Fisher Scientific) at 570 nm with subtraction of the absorbance of the cell-free blank volume at 630 nm. Cell viability was expressed as a percent of the control culture value, and it was calculated using the following equation: Cells viability (%) = (OD dye –ODK dye) / (OD control -ODK control) × 100.

6. Live cell imaging experiments

Human cervical cancer cells (HeLa), Human breast cancer cells (MCF-7), and African green monkey kidney cells (COS-7) were cultured in Dulbecco's modified Eagle's medium (Invitrogen) supplemented with 10 % fetal bovine serum (Invitrogen). Cells were seeded in 24-well flat-bottomed plates and incubated for 24 h at 37 ℃ under 5 % CO_2. **1** (5 μM) was added (the concentration of DMSO was maintained to be less than 0.2 %) and cells were further incubated for 20 min. The fluorescence imaging was performed with OLYMPUS FV-1000 inverted fluorescence microscope with 100×objective lens. Under the confocal fluorescence microscope, **1** was excited at 405 nm and emission was collected at 510-540 nm.

For the thiols-blocking experiment, MCF-7, COS-7 and HeLa cells were precultured with NMM (1 mM) at 37 ℃ for 20 min, and then treated with Cys, Hcy, GSH (100 μM) for another 20 min.

For co-localization experiments, MCF-7 and COS-7 cells were incubated with 1 (5 μM) at 37 ℃ for 20 min, and then treated with Lyso-Tracker Red (1 μM) at 37 ℃ for another 20 min.

7 Tissue imaging experiments

Two-photon fluorescence imaging of **1** in tissues were obtained with a spectral confocal multiphoton microscope (Olympus, FV1000) with a high-performance mode-locked titanium sapphire laser source (MaiTai, Spectra-Physice, USA). Numerical aperture was 1.42 (oil) and 1.30 (sil), respectively. The excitation wavelength was 805 nm and output power was 1230 mW, which corresponded to approximately 10 mW average power in the focal plane. Images were collected at 520-560 nm.

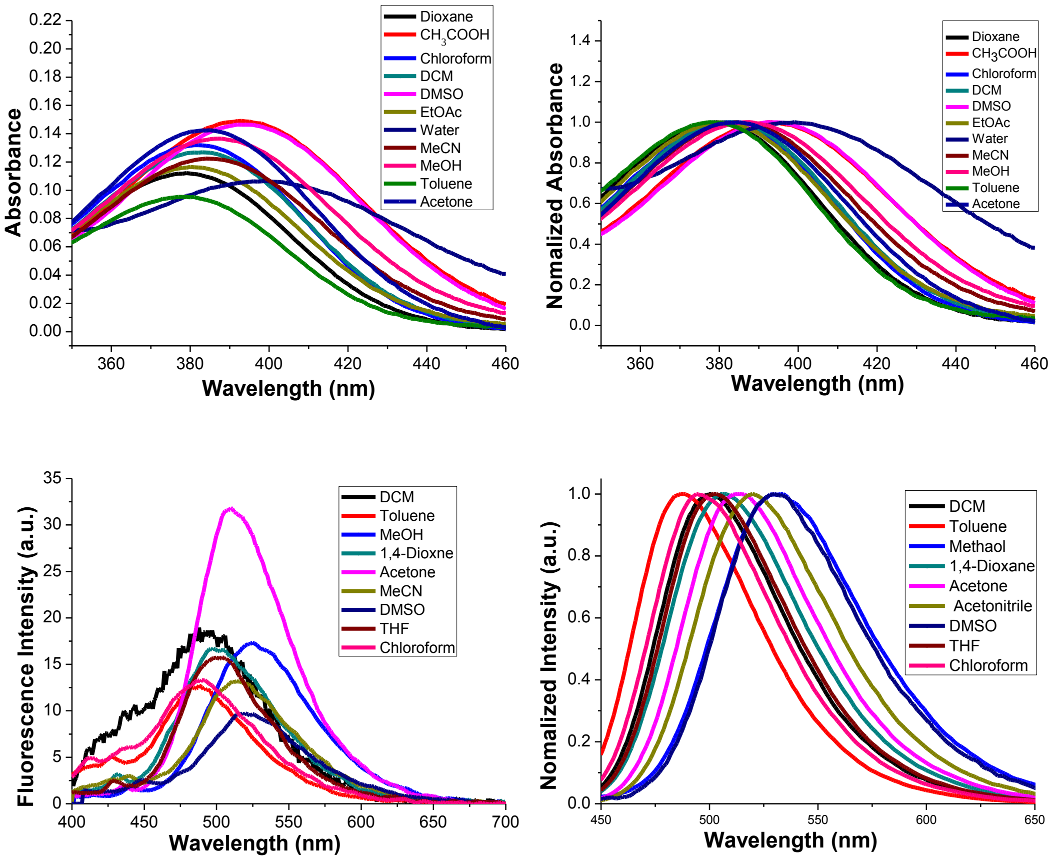

Fig. S1 The absorbance and emission spectra of **1** (10 μM) in different solutions.

Table S1 Photophysical properties of **1** in various solvents.

**Test System**	λ_{abs}[a]/nm	λ_{em}[b]/nm	**Stokes shift**/nm	Φ_F[c]	ε[d]$_{max}$/(mol cm)
Toluene	378	493	115	0.021	3.18×10^4
Chloroform	382	488	106	0.029	4.40×10^4
DCM	383	495	112	0.022	4.23×10^4
1,4-Dioxne	379	493	114	0.004	3.74×10^4
Actone	385	508	176	0.038	4.75×10^4
MeOH	388	518	130	0.015	4.55×10^4
MeCN	384	515	131	0.011	4.08×10^4
DMSO	394	521	127	0.020	4.88×10^4
Water	399	529	130	0.022	3.55×10^4

[a, b] λ_{max} of the absorbance and emission spectra in nm. [c] Fluorescence quantum yield. [d] Molar extinction coefficient.

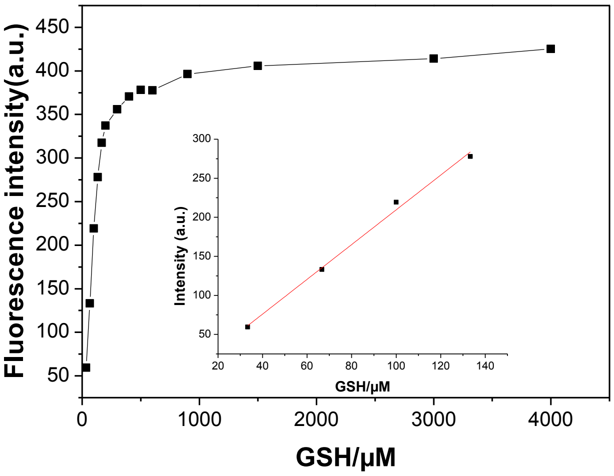

Fig. S2 Fluorescence intensity changes of **1** as a function of concentration GSH (0-200 equiv). Insert: Linear relationship of fluorescence intensity at 540 nm as a function of GSH concentration.

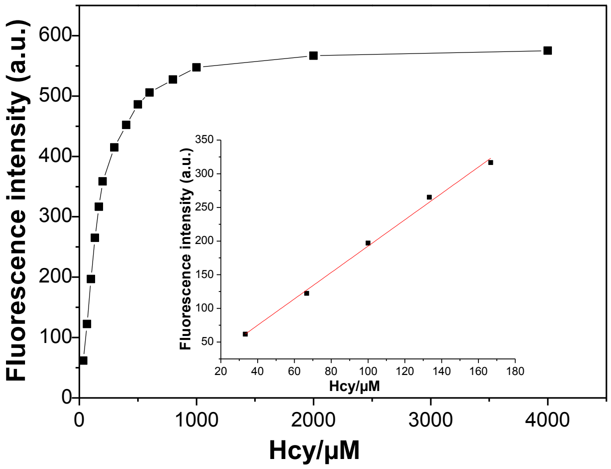

Fig. S3 Fluorescence intensity changes of **1** as a function of concentration Hcy (0-200 equiv). Insert: Linear relationship of fluorescence intensity at 540 nm as a function of Hcy concentration.

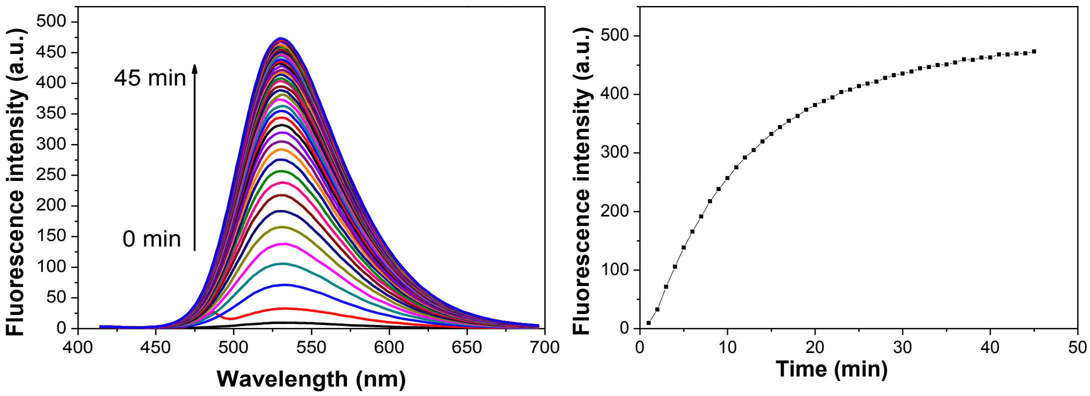

Fig. S4 Time-dependent fluorescence response of **1** (10 μM) in the presence of GSH (100 equiv) in DMSO -HEPES (10 mM, pH 7.4, 3:7, v/v) at 37 ℃.

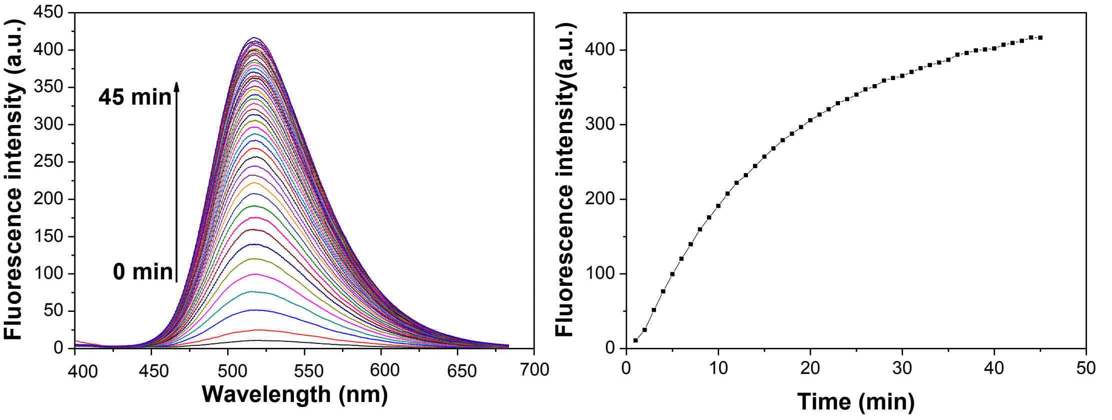

Fig. S5 Time-dependent fluorescence response of **1** (10 μM) in the presence of Hcy (100 equiv) in DMSO -HEPES (10 mM, pH 7.4, 3:7, v/v) at 37 ℃.

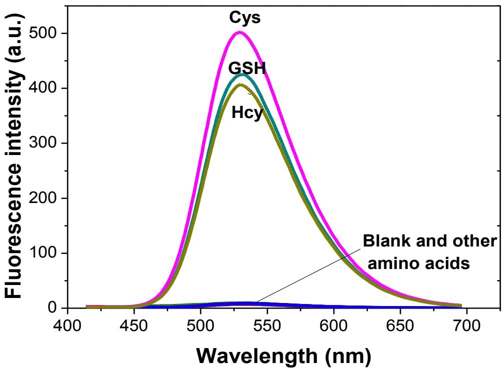

Fig. S6 Fluorescence response of **1** (10 μM) to various amino acids (100 μM). Each spectrum was recorded after 45 min following addition of other amino acids (Gly, Leu, Phe, Ala, Gln, Pro, Val, His, Thr, Lys, Arg, Asn, Glu, Glu, Asp, Tyr, Trp, Ser).

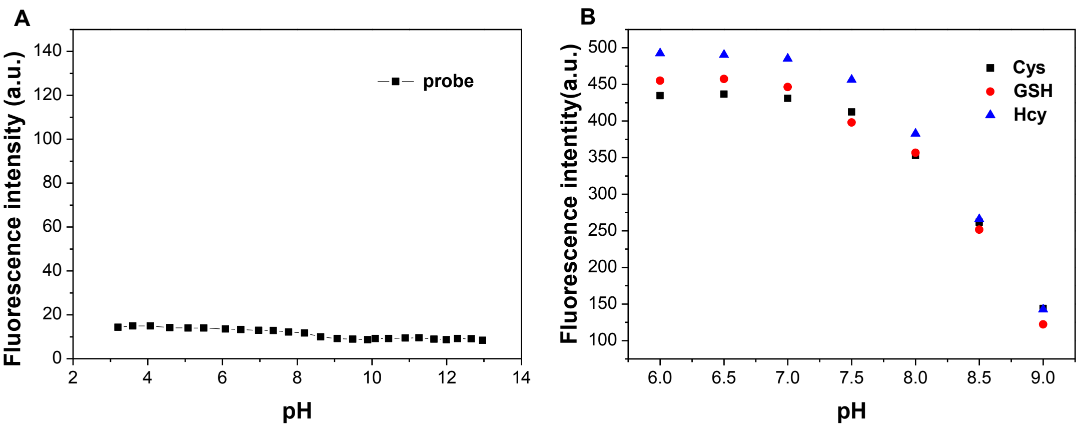

Fig. S7 Effect of pH on the fluorescence intensities of **1** (10 μM) in the absence (A) and presence (B) of thiols (100 equiv).

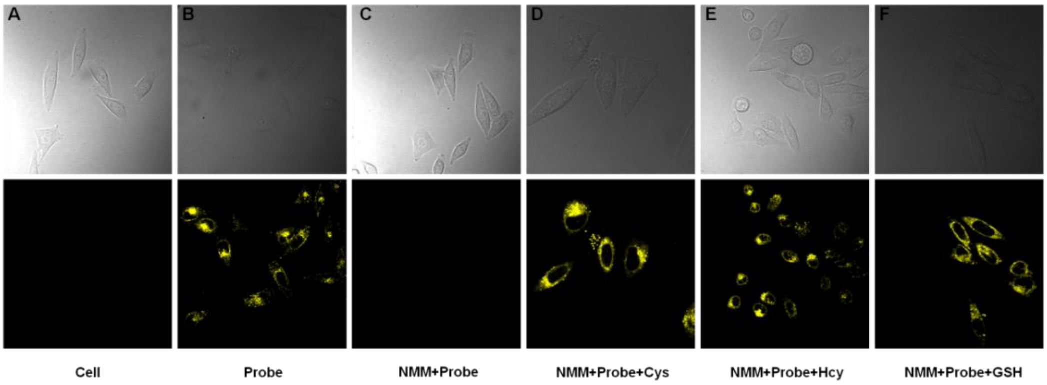

Fig. S8 Top Row: Optical microscopy images of MCF-7 cells (A) untreated; (B) incubated with **1** (5 μM) for 20 minutes; (C) pretreated with NMM (1 mM) followed by incubation with **1**; and (D-F) pretreated with NMM, then treated with Cys, Hcy, or GSH (100 μM), respectively, and finally incubated with **1**. Bottom Row: Fluorescent emission images of the above cell lines. Two-photon excitation was provided at 805 nm with fs pulses, and the TPEF were collected at 520-560 nm. Scale bar: 30μm. Cells are shown representative images from replicate experiments (n = 5).

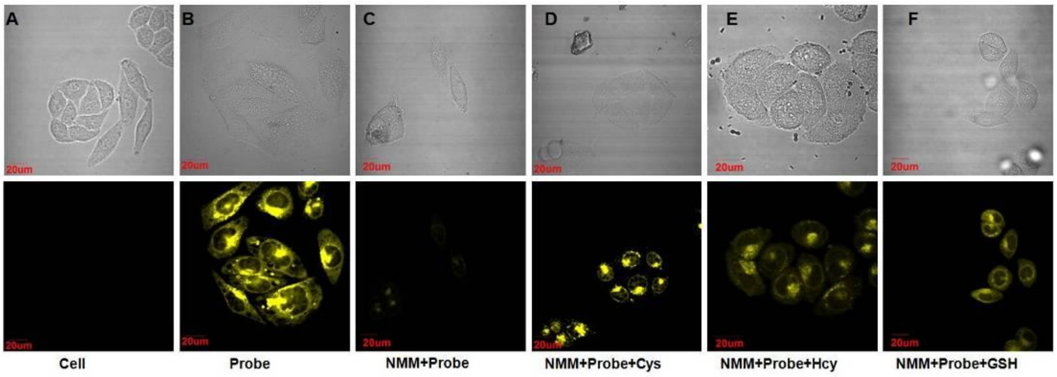

Fig. S9 Top Row: Optical microscopy images of HeLa cells (A) untreated; (B) incubated with **1** (5 μM) for 20 minutes; (C) pretreated with NMM 1(mM)followed by incubation with **1**; and (D-F) pretreated with NMM, then treated with Cys, Hcy, or GSH (100 μM), respectively, and finally incubated with **1**. Bottom Row: Fluorescent emission images of the above cell lines. Emission was collected at 510-540 nm for yellow channel (excited at 405 nm). Cells shown are representative images from replicate experiments (n=5).

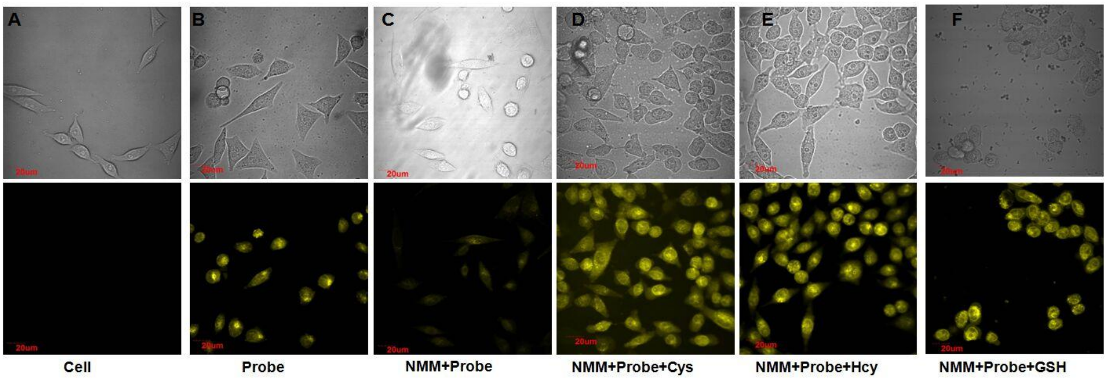

Fig. S10 Top Row: Optical microscopy images of MCF-7 cells (A) untreated; (B) incubated with **1** (5 μM) for 20 minutes; (C) pretreated with NMM (1 mM) followed by incubation with **1**; and (D-F) pretreated with NMM, then treated with Cys, Hcy, or GSH (100 μM), respectively, and finally incubated with **1**. Bottom Row: Fluorescent emission images of the above cell lines. Emission was collected at 510-540 nm for yellow channel (excited at 405 nm). Cells shown are representative images from replicate experiments (n=5).

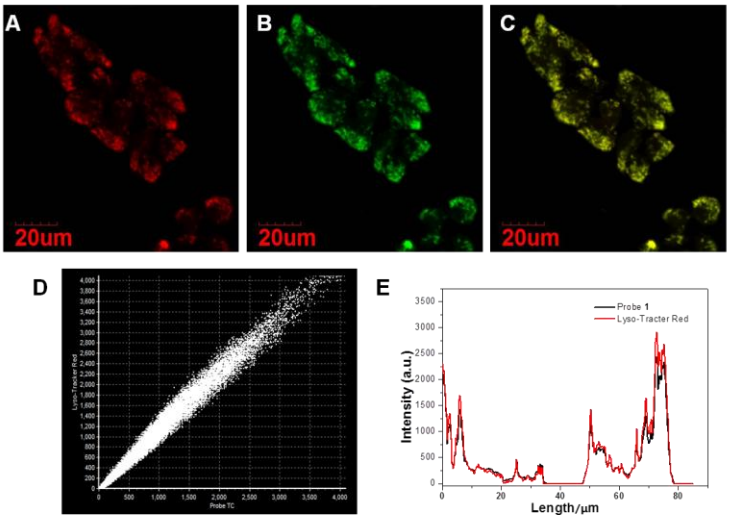

Fig. S11 Co-localization experiments using **1** to lysosomes in COS-7 cells. (A) Cells were stained with **1** (5 μM) for 20 min at 37 ℃ and (B) Lyso-Tracker Red (1.0 μM) for 20 min. (C) overlay of (A) and (B). (D) Intensity correlation plot of stain **1** and Lyso-Tracker Red. (E) Intensity profile of regions of interest (ROI) across COS-7 cells. The excitation wavelengths were 405 nm (**1**) and 559 nm (Lyso-Tracker Red). The fluorescence was collected at 510-540 nm (**1**) and 580–600 nm (Lyso-Tracker Red). Cells shown are representative images from replicate experiments (n=5).

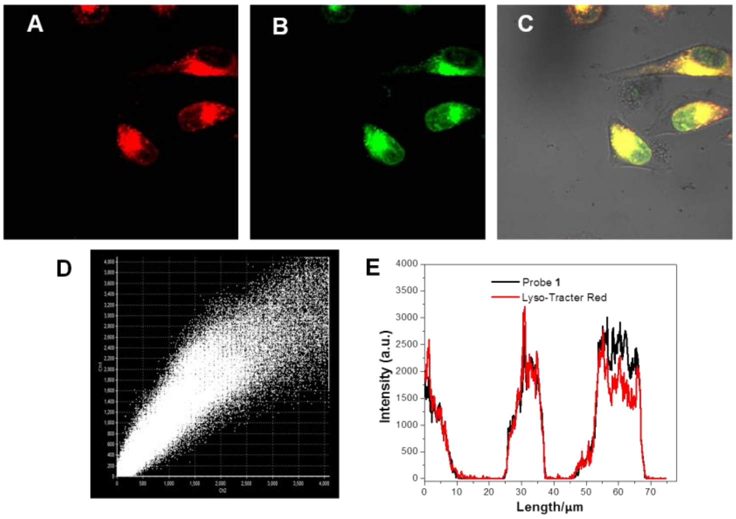

Fig. S12 Co-localization experiments using **1** to lysosomes in MCF-7 cells. Cells were stained with (A) **1** (5 μM) for 20 min at 37 ℃ and (B) Lyso-Tracker Red (1.0 μM) for 20 min. (C) overlay of (A) and (B). (D) Intensity correlation plot of stain **1** and Lyso-Tracker Red. (E) Intensity profile of regions of interest (ROI) across MCF-7 cells. The excitation wavelengths were 405 nm (**1**) and 559 nm (Lyso-Tracker Red). The fluorescence was collected at 510-540 nm (**1**) and 580–600 nm (Lyso-Tracker Red). Cells shown are representative images from replicate experiments (n=5).

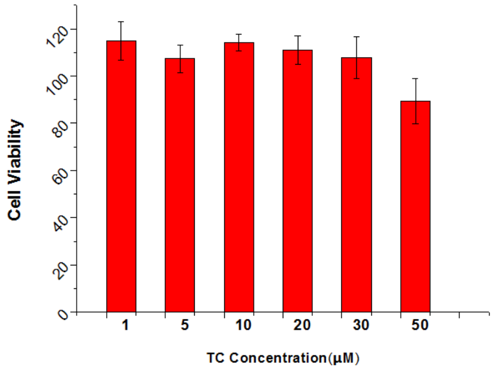

Fig. S13 Biological toxicity of **1** in living MCF-7 cells for 24 h.

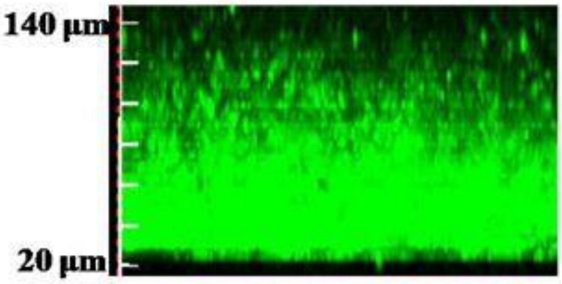

Fig. S14 Depth imaging of **1** (10 μM) in tissues. Images were generated using excitation by the two-photon lasers at 805 nm and were collected at 520−560 nm.

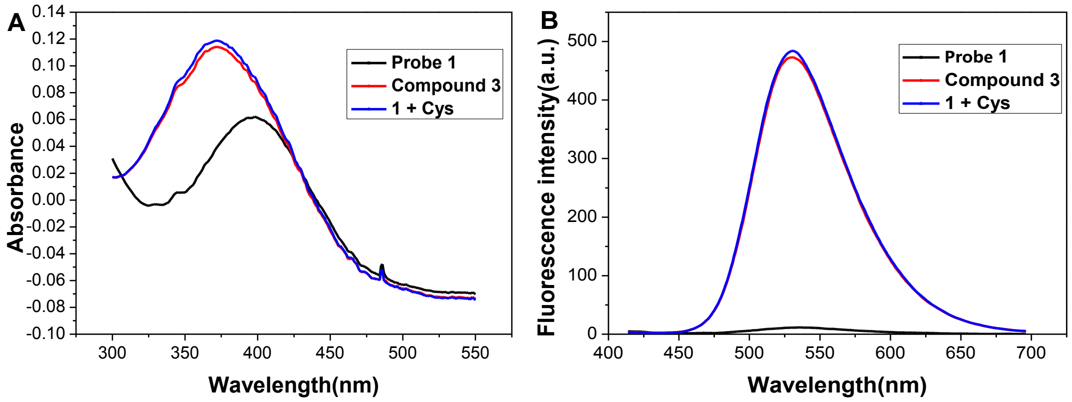

Fig. S15 (A) Absorbance and (B) emission spectra of 10 μM **3** (red line), 10 μM **1** (black line) and 10 μM **1** with 50 μM Cys (blue line) in a mixture of DMSO-HEPES (10 mM, pH 7.4, 3:7, v/v) at 37 ℃, λ_{ex} = 400 nm.

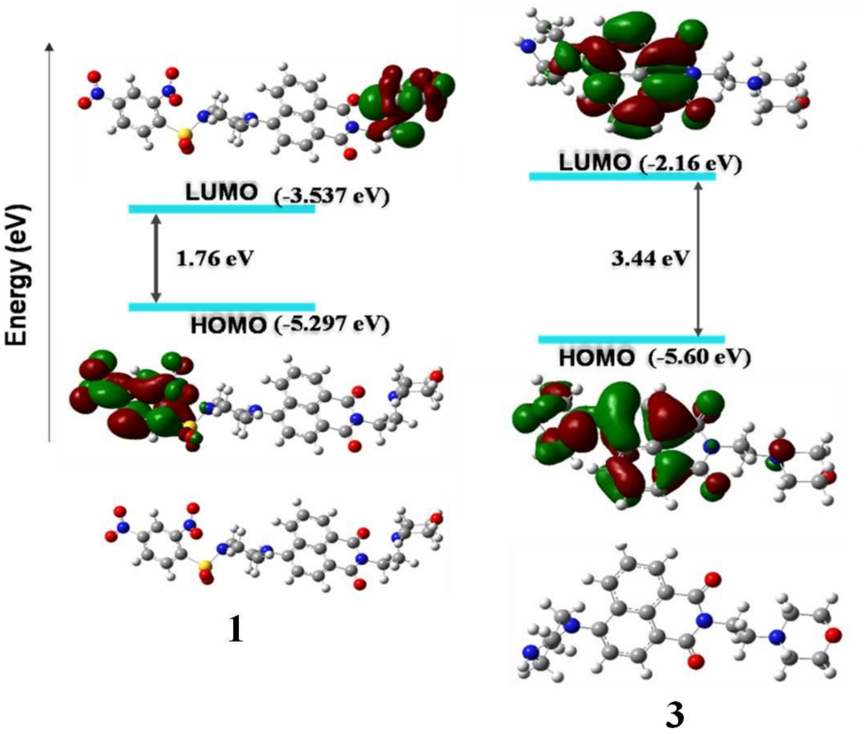

Fig. S16 Frontier molecular orbital profiles of **1** and **3** based on DFT (B3LYP/6-311G(d, p)) calculations.

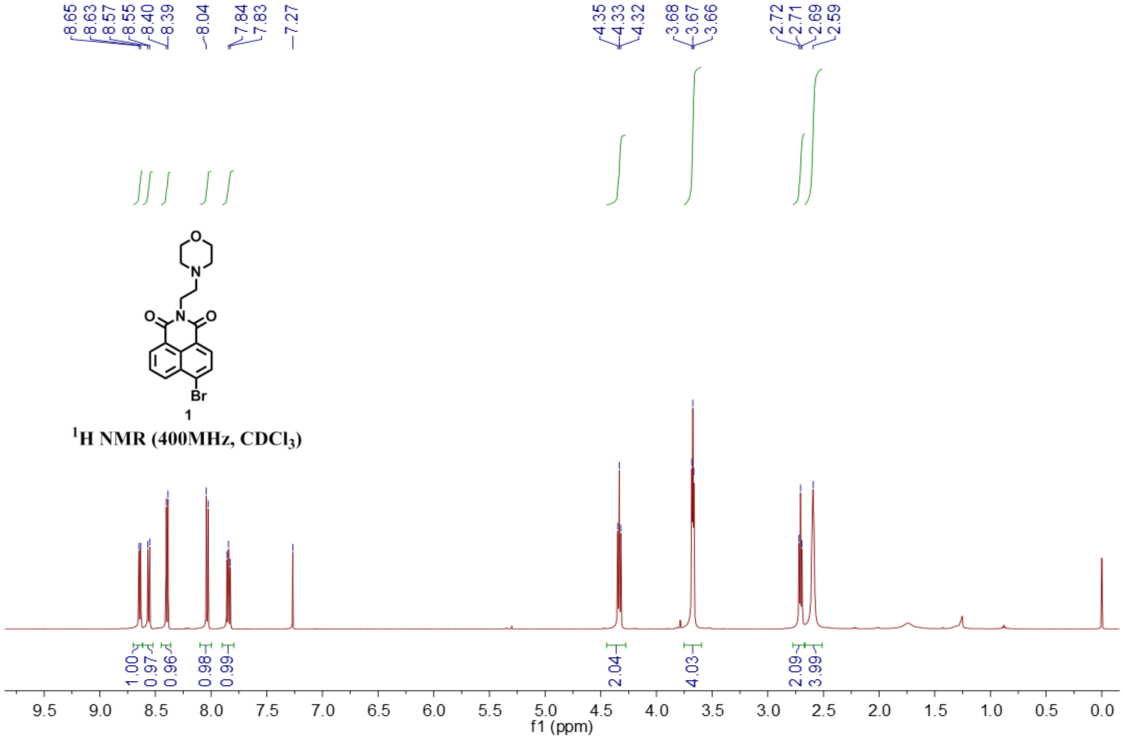

Fig. S17 ^{1}H-NMR spectrum of compound **5** recorded in $CDCl_3$.

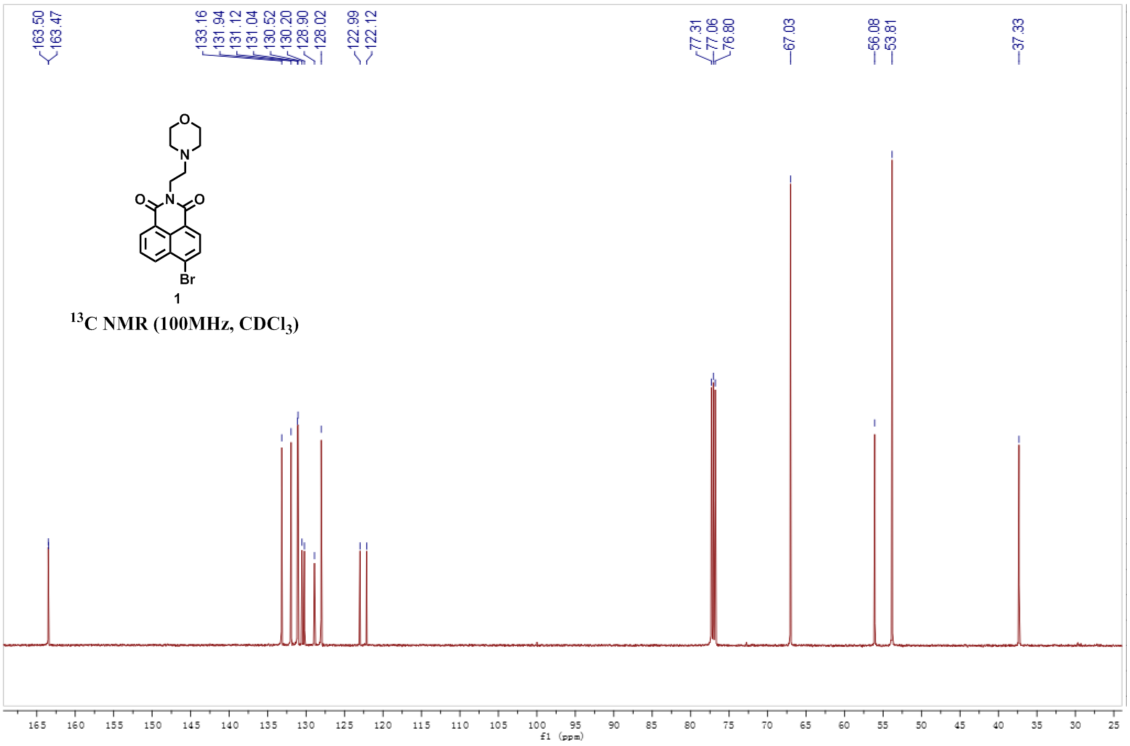

Fig. S18 ^{13}C -NMR spectrum of compound **5** recorded in $CDCl_3$.

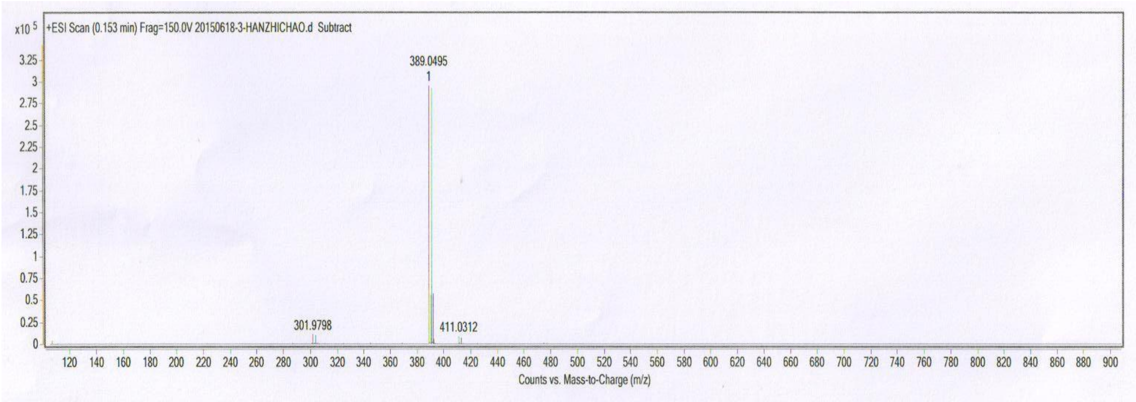

Fig. S19 HR MS of compound **5**.

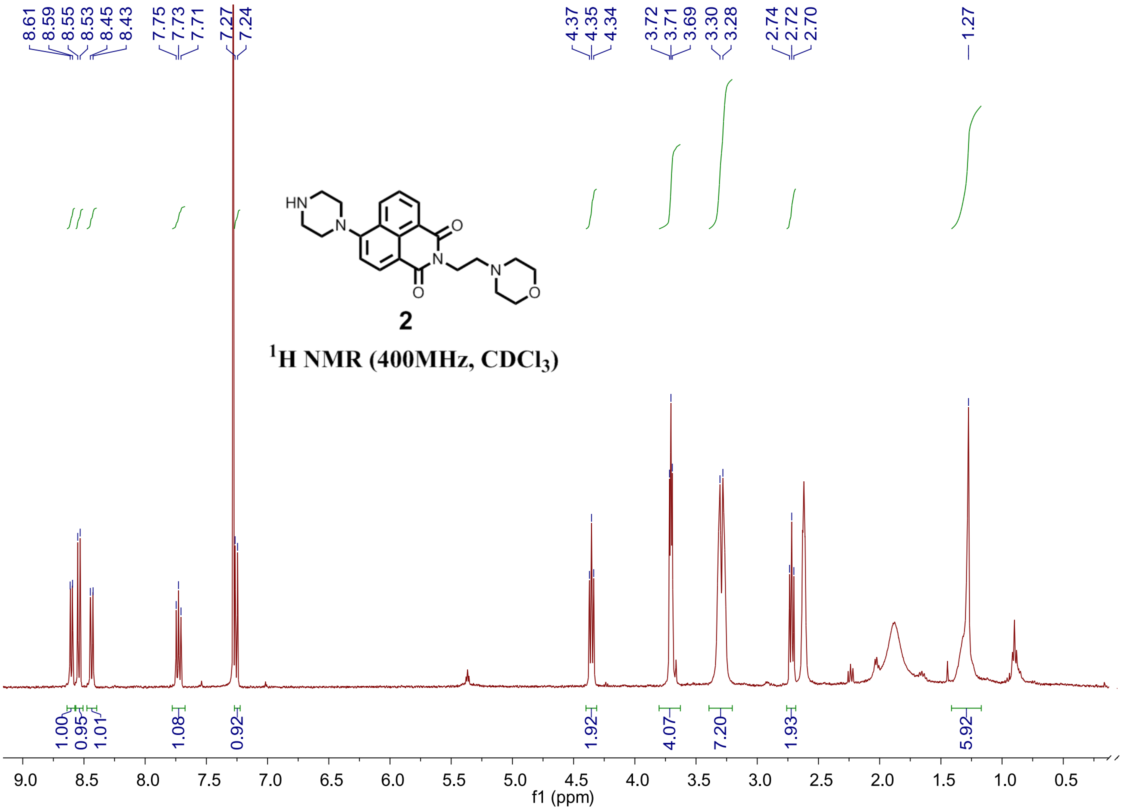

Fig. S20 ^{1}H-NMR spectrum of compound **3** recorded in $CDCl_3$.

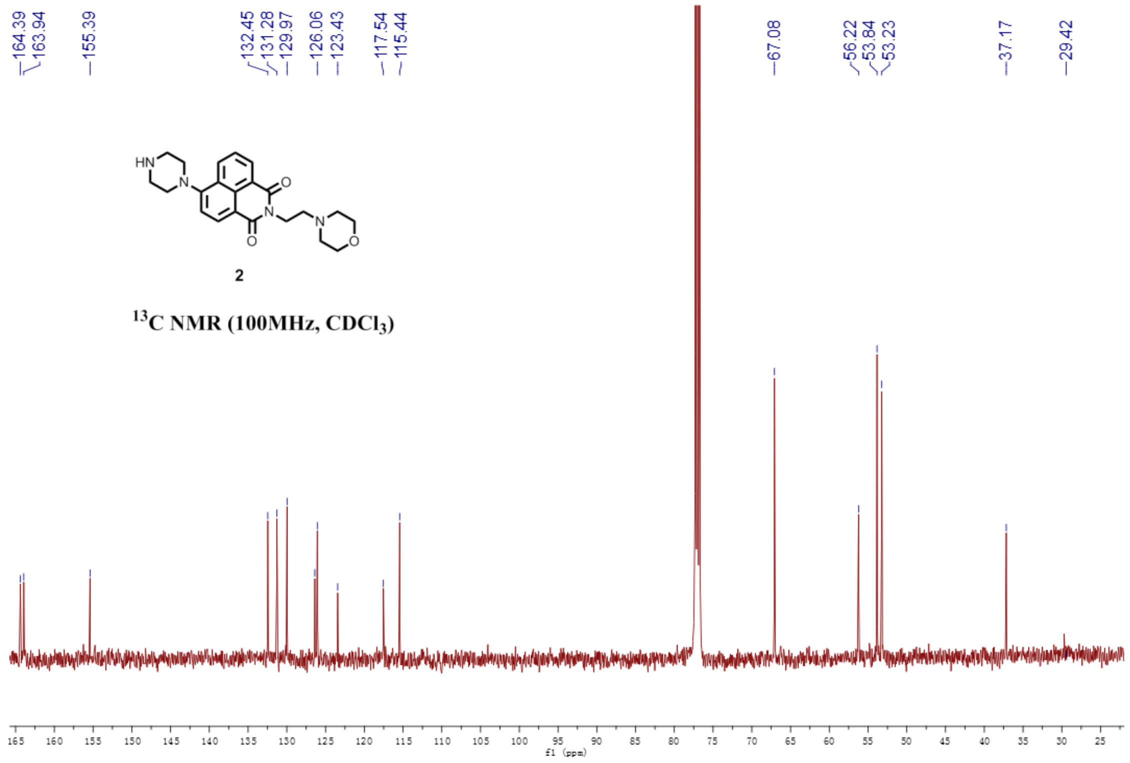

Fig. S21 ^{13}C-NMR spectrum of compound **3** recorded in $CDCl_3$.

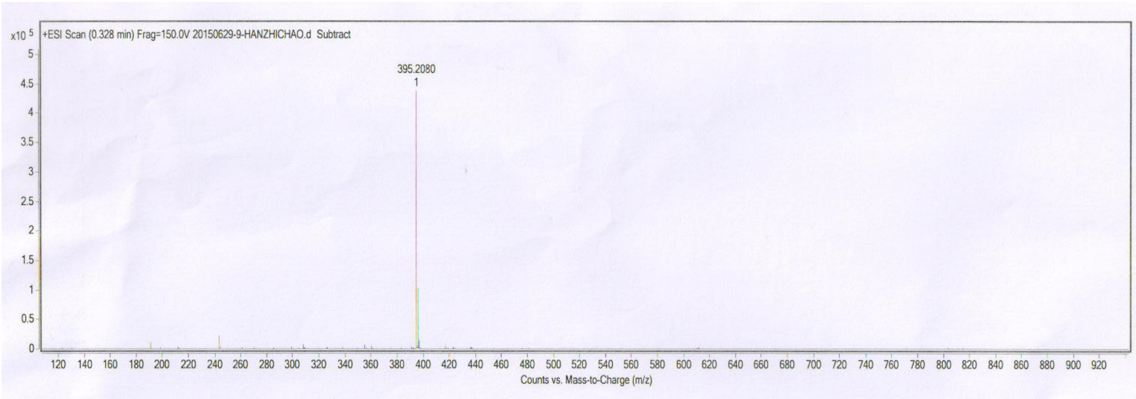

Fig. S22 HR MS of compound **3**.

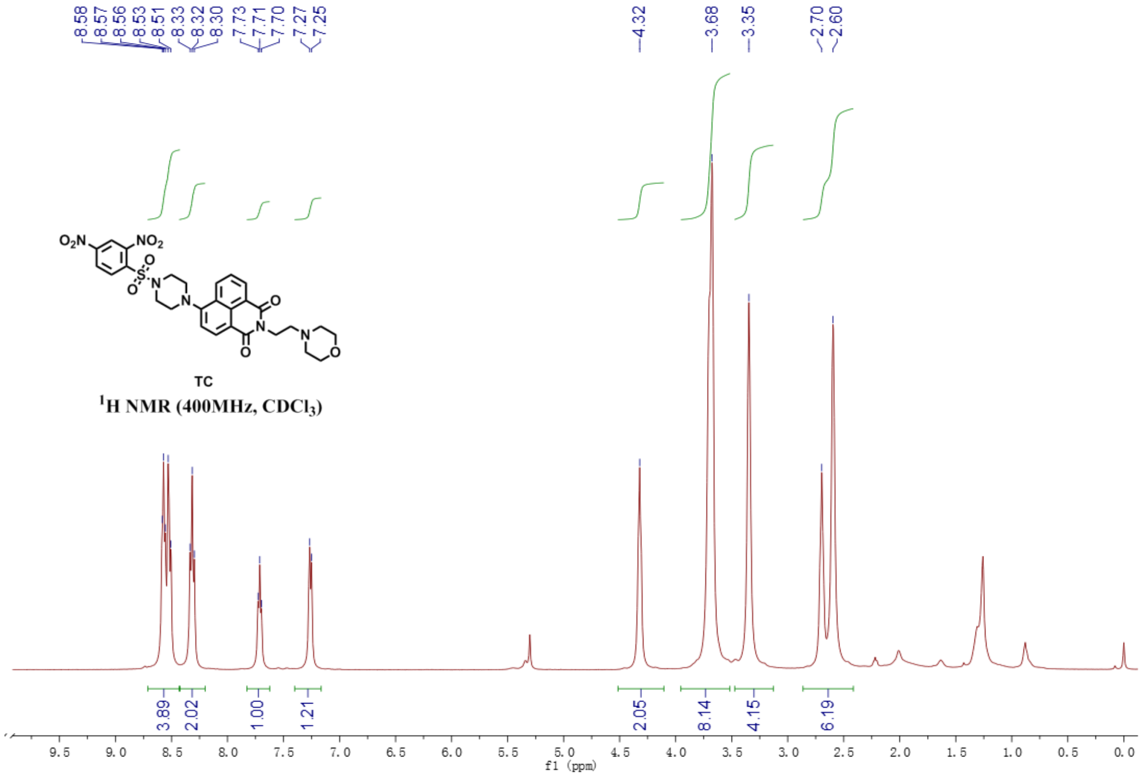

Fig. S23 ^{1}H-NMR spectrum of compound **1** recorded in $CDCl_3$.

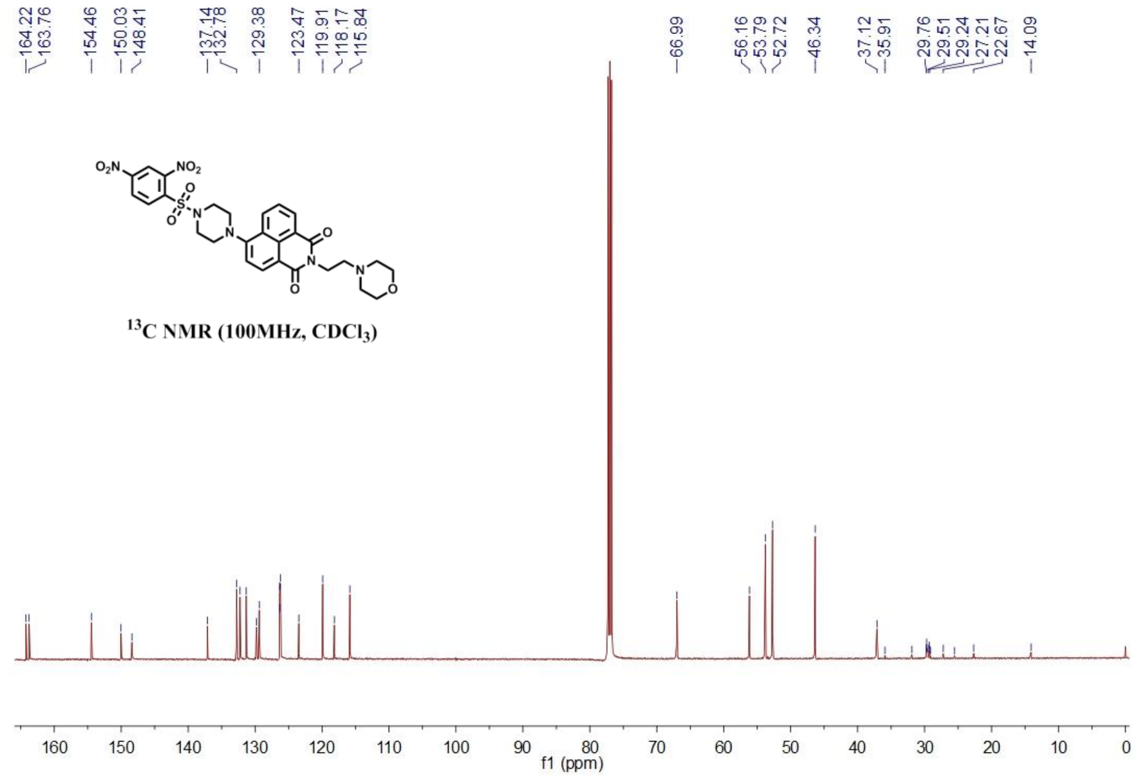

Fig. S24 ^{13}C-NMR spectrum of compound **1** recorded in $CDCl_3$.

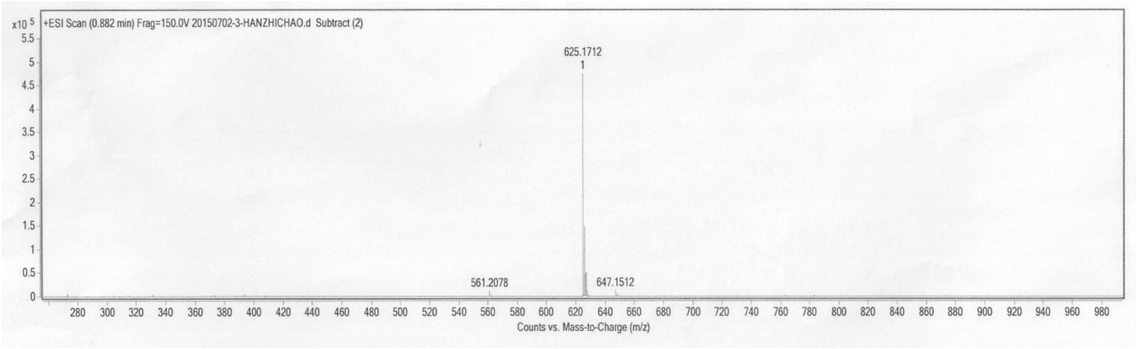

Fig. S25 HR MS of compound **1**.

8 Supplementary references

1. T. Karstens and K. Kobs, *J. Phys. Chem.*, 1980, **84**, 1871-1872.

2. K. G. Casey and E. L. Quitevis, *J. Phys. Chem.*, 1988, **92**, 6590-6594.
